# DIRAS3, GPR171 and RAC2 were identified as the key molecular patterns associated with brain metastasis of breast cancer

**DOI:** 10.3389/fonc.2022.965136

**Published:** 2022-09-21

**Authors:** Ji Dai, Qi Chen, Guoqing Li, Mengze Chen, Haohang Sun, Meidi Yan

**Affiliations:** General Surgery I (Thyroid, Breast, Vascular, Hernia Surgery), People's Hospital of Zhenhai, Ningbo, China

**Keywords:** breast cancer, brain metastasis, bioinformatic analysis, immune cells, DIRAS3

## Abstract

**Objective:**

Brain metastasis is a primary cause of morbidity and mortality in breast cancer patients. Therefore, elucidation and understanding of the underlying mechanisms are essential for the development of new therapeutic strategies.

**Methods:**

Differential gene analysis was performed for those with and without distant metastasis in The Cancer Genome Atlas (TCGA) database and those with and without recurrence in the brain in the dataset GSE12276. The differentially expressed genes procured from the two databases were intersected to obtain the intersecting genes associated with brain metastasis. Thereafter, the intersecting genes were subjected to LASSO model construction to screen for prognostic genes. The expression of the obtained genes in metastatic breast cancer was observed, and survival analysis was performed. Finally, GSEA analysis of the obtained genes was performed, and the relationship between them and immune cells was explored.

**Results:**

A total of 335 differential genes for the occurrence of distant metastases were obtained based on the TCGA database. A total of 1070 differential genes for recurrence to the brain were obtained based on the dataset GSE12276. The Venn diagram showed 24 intersecting genes associated with brain metastasis. The LASSO prognostic model contained a total of five genes (GBP2, GPR171, DIRAS3, RAC2, and CACNA1D). Expression difference analysis showed that GBP2, GPR171, DIRAS3, and RAC2 were significantly down-regulated in expression in metastatic breast cancer compared with primary breast cancer tumors. Only GPR171, DIRAS3, and RAC2 were strongly correlated with the overall survival of breast cancer patients. Their correlation analysis with immune cells showed that the correlation coefficient between the expression levels of DIRAS3 and immune cells was low, and the expression levels of GPR171 and RAC2 were more closely correlated with B cells and macrophages.

**Conclusions:**

The expression of DIRAS3, GPR171 and RAC2, genes associated with brain metastasis, was reduced in metastatic breast cancer, and GPR171 was found to promote brain metastasis of breast cancer cells by inducing B cells and thereby.

## Introduction

Breast cancer is a life-threatening disease and a major cause of death in women. Among all malignant diseases, breast cancer is considered to be one of the leading causes of death in postmenopausal women (1). Data show that 8 million people died from malignant diseases in 2008, and this number is expected to reach 11 million by 2030 (2). Depending on the cellular origin involved, breast cancer can be divided into two main categories: carcinoma and sarcoma. Carcinoma accounts for the majority of breast cancers, while sarcomas, such as lobular tumors and angiosarcomas, are rare. Carcinoma is a breast cancer caused by the epithelial component of the breast, which consists of cells arranged in lobules and terminal ducts (3). Over the past two decades, research related to breast cancer has led to astonishing advancements in our understanding of breast cancer, resulting in further skilled treatments. However, women are still diagnosed at advanced stages due to negligence in self-examination of the breast and clinical examination (4).

Advanced breast cancer is usually metastatic, and metastases can be found in axillary lymph nodes or distant sites, such as the lung, liver, bone, and brain. Distant metastasis is a complex multistep process in which tumor cells detach from the primary tumor, infiltrate the body circulation, survive in circulation, evade immune attack, adhere to capillaries, and exude before they colonize distant organs (5). Even after the primary tumor is removed, tiny tumor cells or microscopic metastases may remain in the body, allowing cancer to recur and spread in more than 30% of breast cancer patients (6). The bone is the most affected metastatic site, while the brain is the least affected metastatic site (7). Nevertheless, brain metastases from breast cancer require attention. Although there are several available treatments for brain metastases, such as chemotherapy, radiotherapy, and targeted therapy, the survival rate of breast cancer patients with brain metastases remains low. Therefore, elucidating and understanding the underlying mechanisms are essential for the development of new therapeutic strategies.

## Methods

### Data collection

Raw counts of all breast cancer RNA sequencing data and corresponding clinical information were obtained from TCGA database (https://portal.gdc.com). Samples were grouped according to clinical information with or without distant metastasis, of which 22 samples developed distant metastasis (M1) and 907 samples did not (M0).

The dataset GSE12276 microarray raw data were downloaded from the GEO database (http://www.ncbi.nlm.nih.gov/geo/). These contained gene expression profiles and 204 breast cancer samples with 188 samples without recurrence in the brain and 16 samples with recurrence in the brain.

### Differential gene analysis

Differentially expressed genes with or without distant metastasis and with or without recurrence in the brain were identified in both databases using the R package limma method, with |log2 FC|<0.3785, p<0.05 as the differential gene screening threshold. The Venn diagram shows the intersection of genes that metastasize and recur to the brain.

### Prognostic modeling

Feature selection was performed using the R package glmnet for the minimum absolute shrinkage and the LASSO regression algorithm using 10-fold cross-validation. For Kaplan–Meier curves, log-rank tests and univariate Cox proportional hazards regression were derived and included p-values and hazard ratios (HRs) with 95% confidence intervals (CIs). TimeROC analysis was used to compare the predictive accuracy and risk scores of the genes. All of the above analyses were performed using the R package. P< 0.05 was considered statistically significant.

### Kaplan–Meier survival curve analysis

Survival analysis was performed using the Kaplan–Meier plotter (kmplot.com/analysis). To assess the prognostic values of specific genes, patient samples were divided into two clusters (high versus low expression) based on the median expression of the genes. Log-rank p-values and HR with 95% CIs were determined. P< 0.05 was considered statistically significant.

### GSEA analysis

Samples were divided into high and low expression groups based on the median gene expression values, and then enrichment analysis of the KEGG pathway was performed using GSEA. FDR< 0.25, NOM p-value< 0.05 and |NES|> 1 were considered significant enrichment. The top three ranked signaling pathways were selected for this study.

## Results

### Differential gene analysis

**Figure 1:**
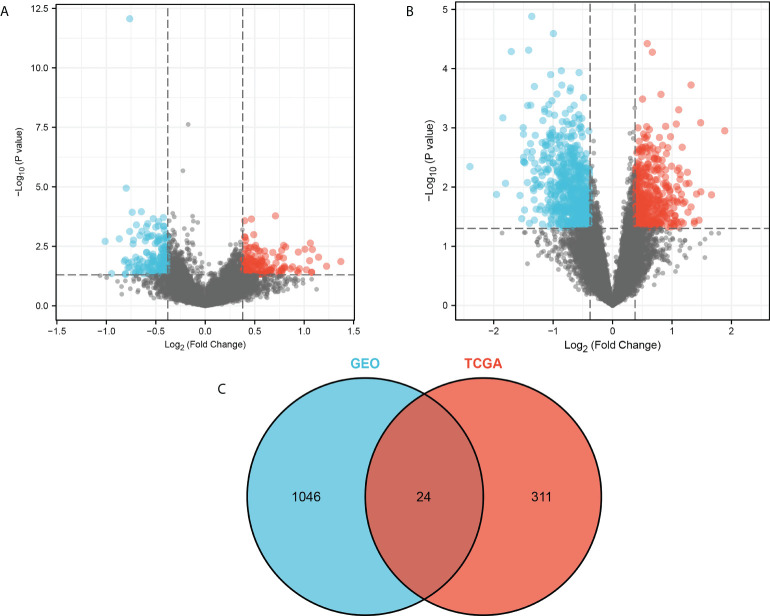
Differential gene analysis. **(A)** Differential gene analysis based on TCGA database is shown with a volcano plot. **(B)** Differential gene analysis based on the GEO dataset GSE12276 is shown with a volcano plot. **(C)** Venn diagram showing intersecting genes. The blue color in the volcano plot indicates down-regulated genes, while the red color indicates up-regulated genes.

We first performed differential gene analysis for breast cancer patients with or without distant metastasis based on TCGA database, with |log2 FC|<0.3785, p< 0.05 as the screening threshold and obtained a total of 335 differential genes, including 163 up-regulated and 172 down-regulated genes. In addition, we performed differential gene analysis for breast cancer with or without recurrence in the brain in the GEO dataset GSE12276 using |log2 FC|<0.3785, p<0.05 as the screening threshold and obtained a total of 1070 differential genes, including 403 up-regulated and 667 down-regulated genes. The Venn diagram shows 24 intersecting genes associated with brain metastasis (see **Figure 1**).

### Prognostic model construction

We then constructed the prognostic model based on LASSO for the 24 genes obtained from the intersection. A RiskScore formula with five genes was obtained as follows:


RiskScore=(−0.0164)∗GBP2 + (−0.0589)*GPR171+(−0.0187) ∗ DIRAS3+(−0.085) ∗ RAC2+(−0.0379)∗CACNA1D

**Figure 2:**
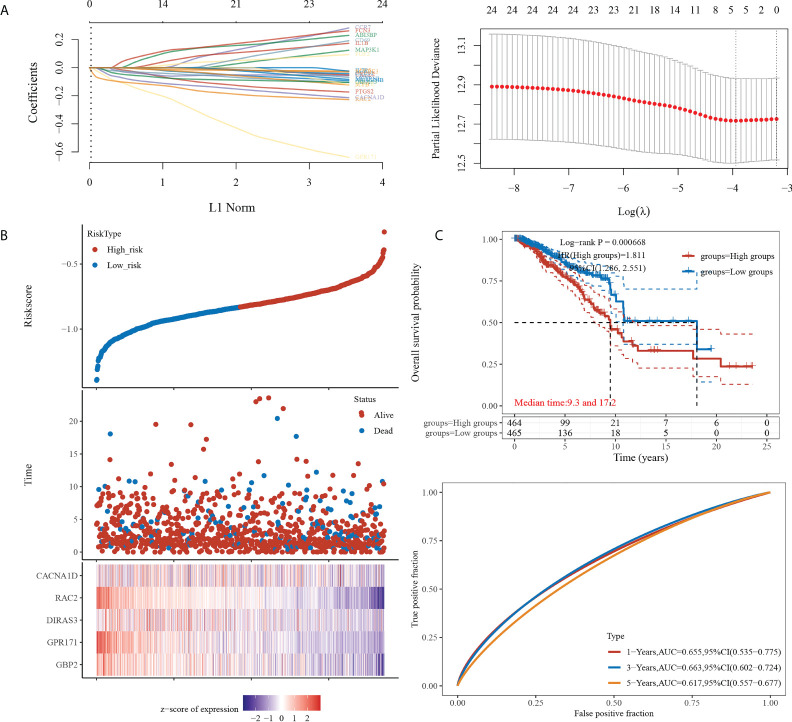
Prognostic model construction. **(A)** (Left) Lambda parameters are shown, with the horizontal axis representing the value of the independent variable lambda and the vertical axis representing the coefficient of the independent variable. (Right) Partial likelihood deviation versus log(λ). **(B)** (Upper) Scatterplot of RiskScore from low to high. (Middle) Scatterplot distribution of survival time and survival status corresponding to different sample RiskScores. (Lower) Heat map of gene expression in this model. **(C)** (Upper) Distribution of KM survival curves of this model in TCGA dataset. (Lower) ROC curves with AUC for one, three, and five years of this model.

The analysis showed that the prognosis of low expression of the gene in this model was significantly better than that of high expression. The AUC areas of this model were 0.655, 0.663, and 0.617 at one, three, and five years, respectively (see **Figure 2**).

### Expression difference analysis

**Figure 3:**
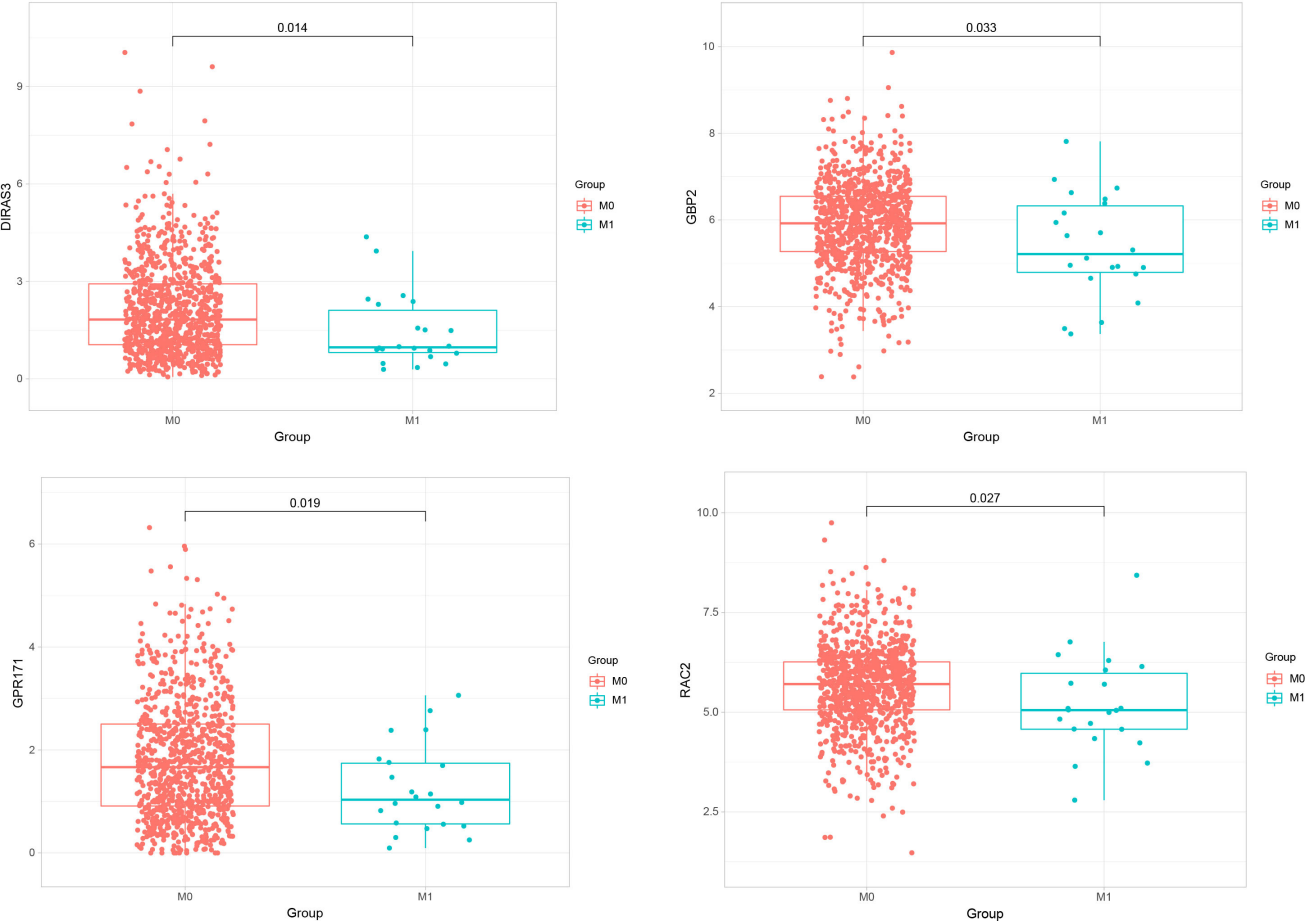
Expressions of GBP2, GPR171, DIRAS3, and RAC2 in primary breast cancer and metastatic breast cancer.

We performed expression analyses of five genes (GBP2, GPR171, DIRAS3, RAC2, CACNA1D) obtained from the LASSO model to observe their expressions in primary breast cancer and metastatic breast cancer. The analysis showed that only GBP2, GPR171, DIRAS3, and RAC2 were significantly down-regulated in expression in metastatic breast cancer compared with primary breast cancer tumors (see **Figure 3**).

### KM survival analysis

**Figure 4:**
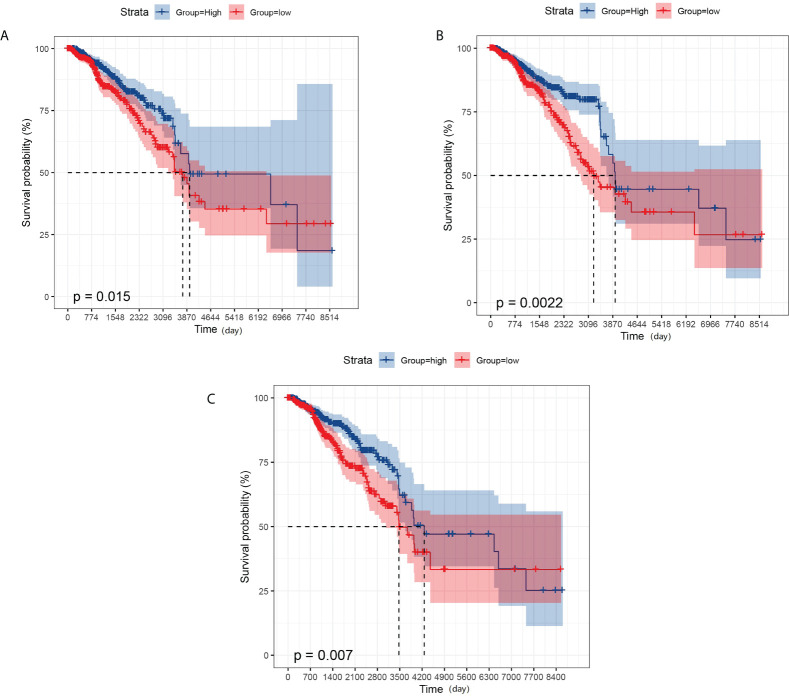
KM survival analysis. **(A)** Effect of the expression level of GPR171 on overall survival of breast cancer patients. **(B)** Effect of the expression level of RAC2 on overall survival of breast cancer patients. **(C)** Effect of the expression level of DIRAS3 on overall survival of breast cancer patients.

We also performed a prognostic analysis of genes that have significant differences in expression in metastatic breast cancer. The analysis showed that GBP2 was not significantly different from the overall survival of breast cancer patients, while GPR171, DIRAS3, and RAC2 were strongly associated with the overall survival of breast cancer patients (see **Figure 4**).

### GSEA analysis

**Figure 5:**
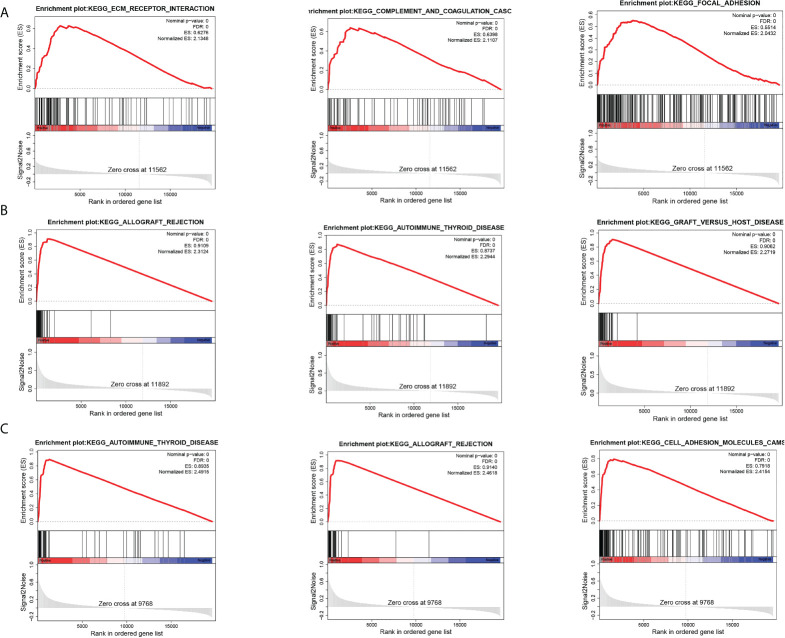
GSEA analysis. **(A)** Top three pathways enriched in DIRAS3 high-expression phenotype. **(B)** Top three pathways enriched in GPR171 high-expression phenotype. **(C)** Top three pathways enriched in RAC2 high-expression phenotype.

To further explore the biological pathways most relevant to the pathogenesis of breast cancer brain metastasis, we performed GSEA analysis on GPR171, DIRAS3, and RAC2. FDR< 0.25, NOM p-value< 0.05, and |NES|> 1 were considered to be significantly enriched. Analysis revealed that the gene sets associated with ECM-receptor interactions, complement and cohesion cascades, and adherent spot signaling were differentially enriched in the DIRAS3 high-expression phenotype. Moreover, genomes associated with allograft rejection, autoimmune thyroid disease, and graft-versus-host disease signaling were differentially enriched in the GPR171 high-expression phenotype. Genomes associated with autoimmune thyroid disease, allograft rejection, and cell adhesion molecule signaling were differentially enriched in the RAC2 high-expression phenotype (see **Figure 5**).

### Gene and immune cell correlation analysis

**Figure 6:**
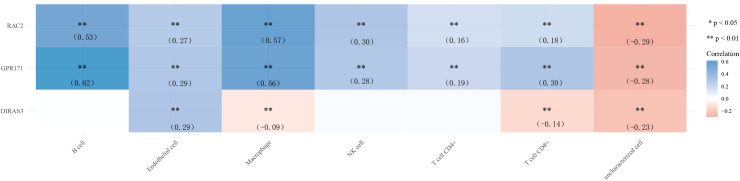
Heat map of correlation between the expression levels of DIRAS3, GPR171, and RAC2 and immune cells. * p< 0.05, ** p< 0.01.

Finally, we explored the correlation between gene expression levels and immune cells. The results of the correlation heat map showed that DIRAS3 expression levels were weakly correlated with the levels of multiple immune cells, while the expression levels of GPR171 and RAC2 were only moderately correlated with the levels of B cells and macrophages. Among them, GPR171 had the highest correlation coefficient with B cells (R=0.62) (see **Figure 6**).

## Discussion

Metastasis is a multistep process that requires uncontrolled tumor growth, penetration of the basement membrane, and new angiogenesis. In the bloodstream, circulating tumor cells extravasate into distant organs by penetrating the endothelium or, in the case of brain metastases, the blood–brain barrier (BBB), to form small colonies in the target organ, survive the apoptotic trail, and finally regrow at secondary sites (8). Brain metastases are a primary cause of morbidity and mortality in breast cancer patients. In autopsy studies, 15% to 35% of breast cancer patients are found to have brain metastases, and not all of these have clinical manifestations prior to death (9, 10). Brain metastases are critical to patient survival, with a median survival of approximately 15 months after diagnosis of brain metastases in breast cancer patients (11). Therefore, unraveling the molecular mechanisms of metastasis formation is vital for the formulation of potential therapeutic interventions.

GPR171 is a GPCR receptor that is closely related to the P2Y receptor, a group of GPCR receptors known to be important for immune response (12). It has been shown that GPR171 is a pro-oncogene that induces proliferation, invasion, and migration of tumor cells (13). DIRAS3 is an oncogene involved in tumor development and autophagy, and low expression of DIRAS3 is associated with high malignancy in ovarian, breast, and prostate cancers (14). Furthermore, in ovarian and breast cancers, DIRAS3 inhibits cell migration, induces autophagy, and increases sensitivity to chemotherapy (15, 16). RAC2 is a GTPase with a molecular weight of 21 kDa; it contains the catalytic subunit of NADPH oxidase (17). In addition, RAC2 has been shown to be associated with epithelial cell polarization, a process that is closely related to changes in intercellular junctions, cytoskeletal distribution, and organelle repositioning (18). It has been reported that RAC2 may play a key role in the regulation of the actin cytoskeleton during breast cancer metastasis and that its downregulation is associated with the invasive and metastatic capacities of human cancers (19, 20). All of the foregoing could indicate that DIRAS3, GPR171, and RAC2 play important roles in tumorigenesis and tumor progression. Notably, in the present study, our results also showed that DIRAS3, GPR171, and RAC2 were less expressed in tumors that developed metastatic disease compared with primary breast cancer tumors. Moreover, the expression levels of DIRAS3, GPR171, and RAC2 were strongly correlated with the overall survival of breast cancer patients. Unfortunately, the expression levels of DIRAS3, GPR171, and RAC2 were not found to be significantly associated with the overall survival of patients with metastatic breast cancer, probably because the sample size was small and, therefore, did not show significance. In addition, GSEA analysis showed that DIRAS3, GPR171, and RAC2 are involved in signaling pathways such as ECM–receptor interaction, complement and cohesion cascades, adhesion plaques, allograft rejection, autoimmune thyroid disease, graft-versus-host disease, and cell adhesion molecules.

Tumor cells require considerable potential to multiply and transform into large tumors. The immune system usually tries to identify cancer cells and deoxyribonucleic acid-damaged cells and destroys them (21). Breast cancer may be the result of a malfunction of this useful immune defense and surveillance. Therefore, in the present study, we also analyzed the correlation between the expression levels of DIRAS3, GPR171, and RAC2 and immune cells. The results showed that the correlation between the expression levels of DIRAS3 and immune cells did not show the desired results. However, the expression levels of GPR171 and RAC2 correlated more closely with B cells and macrophages. Among them, GPR171 had the highest correlation coefficient with B cells. It has been indicated that GPR171 expression is inducible in T cells and suppresses T cell-mediated immune responses through GPR171 signaling. Furthermore, the disruption of GPR171 signaling promoted T cell-mediated antitumor immunity (22). It is known that disruption of the Blood Brain Barrier by CNS tumors and changes in the composition of the extracellular matrix can allow leakage of Blood Brain Barrier at the tumor site (23). An intact brain contains almost no lymphocytes, but some studies have shown that T and B cells have been observed in the setting of brain metastases (24). Therefore, we speculate that GPR171 could promote brain metastasis of breast cancer cells by inducing B cells, but the exact mechanism remains to be investigated.

In conclusion, the expressions of DIRAS3, GPR171, and RAC2 genes associated with brain metastasis were reduced in metastatic breast cancer and were strongly associated with overall breast cancer survival. Our study also showed that the expression level of GPR171 was significantly correlated with B cells, suggesting that GPR171 could promote brain metastasis of breast cancer cells by inducing B cells. Our findings lay the foundation for understanding the molecular basis of breast cancer metastasis to distant sites, which will have the potential to contribute to future therapeutic research and provide new directions for exploring new drugs or therapies for breast cancer.

## Data availability statement

The original contributions presented in the study are included in the article/supplementary material. Further inquiries can be directed to the corresponding author.

## Author contributions

JD: Conceptualization, Methodology, Writing - Original Draft. QC: Methodology, Formal analysis, Writing - Review & Editing. GL: Writing - Review & Editing, Visualization, Investigation. MC: Data Curation, Methodology, Writing - Original Draft. HS: Writing - Review & Editing, Formal analysis, Resources. MY: Writing - Original Draft, Funding acquisition, Resources.

## Funding

This work was supported by Ningbo Natural Science Foundation Project (2019A610302) and Zhejiang Province Medical and Health Clinical Research Application Project (2022KY1173).

## Conflict of interest

The authors declare that the research was conducted in the absence of any commercial or financial relationships that could be construed as a potential conflict of interest.

## Publisher’s note

All claims expressed in this article are solely those of the authors and do not necessarily represent those of their affiliated organizations, or those of the publisher, the editors and the reviewers. Any product that may be evaluated in this article, or claim that may be made by its manufacturer, is not guaranteed or endorsed by the publisher.
